# Differential impact of thermal and physical permafrost disturbances on High Arctic dissolved and particulate fluvial fluxes

**DOI:** 10.1038/s41598-020-68824-3

**Published:** 2020-07-16

**Authors:** C. R. Beel, S. F. Lamoureux, J. F. Orwin, M. A. Pope, M. J. Lafrenière, N. A. Scott

**Affiliations:** 1 Department of Geography and Planning, Queen’s University, Kingston, ON K7L 3N6 Canada; 2 Yellowknife Research Office, Wilfrid Laurier University, Yellowknife, NT X1A 2P8 Canada; 3 Resource Stewardship Division, Alberta Environment and Parks, Government of Alberta, Calgary, AB T2L 2K8 Canada

**Keywords:** Carbon cycle, Hydrology

## Abstract

Climate warming and changing precipitation patterns have thermally (active layer deepening) and physically (permafrost-thaw related mass movements) disturbed permafrost-underlain watersheds across much of the Arctic, increasing the transfer of dissolved and particulate material from terrestrial to aquatic ecosystems. We examined the multiyear (2006–2017) impact of thermal and physical permafrost disturbances on all of the major components of fluvial flux. Thermal disturbances increased the flux of dissolved organic carbon (DOC), but localized physical disturbances decreased multiyear DOC flux. Physical disturbances increased major ion and suspended sediment flux, which remained elevated a decade after disturbance, and changed carbon export from a DOC to a particulate organic carbon (POC) dominated system. As the magnitude and frequency of physical permafrost disturbance intensifies in response to Arctic climate change, disturbances will become an increasingly important mechanism to deliver POC from terrestrial to aquatic ecosystems. Although nival runoff remained the primary hydrological driver, the importance of pluvial runoff as driver of fluvial flux increased following both thermal and physical permafrost disturbance. We conclude the transition from a nival-dominated fluvial regime to a regime where rainfall runoff is proportionately more important will be a likely tipping point to accelerated High Arctic change.

## Introduction

Fluvial fluxes of dissolved and particulate material from Arctic watersheds are strongly controlled by the presence of permafrost. Arctic fluvial systems have adapted to strong seasonality in flow generation and relatively low inputs of permafrost-derived terrestrial material (e.g., sediment, carbon), with downstream aquatic ecosystems also adapted to this type of low energy regime^1–2^. However, Arctic fluvial systems are changing due to permafrost disturbance caused by climate warming and shifting precipitation patterns^3–6^. Coupled climate-terrestrial models consistently predict widespread permafrost thaw and disturbance over the next century^7^. Multiple pan-Arctic studies show that observed climate change is already transferring significant quantities of terrestrial material to downstream aquatic ecosystems^8–10^ as a physical response to increases in the magnitude, frequency, and type of permafrost disturbance^11–14^.

Several types of widely observed permafrost disturbance can be broadly grouped into two main disturbance types: thermal and physical^15^. Thermal disturbance refers to climatic conditions that alter the extent of soil thaw and/or active layer depth (e.g., above average summer air temperatures), usually during one or multiple thaw seasons. Thermal disturbances may have little geomorphic expression on the land surface, but a measurable impact on surface water quality as sub-surface drainage connectivity changes with increased thaw and active layer depths^16–17^. Physical disturbance refers to permafrost-thaw related mass movements that displace or rearrange the physical properties of the active layer, either permanently or in an ongoing manner. Physical disturbances visibly change terrestrial surfaces, altering watershed hydrology and rapidly mobilizing significant quantities of dissolved and particulate material to downstream aquatic environments^8,9^.

Disturbance of permafrost landscapes are predicted to have a profound impact on regional and global carbon (C) cycles^18–20^. Permafrost landscapes are estimated to store 1,035 ± 150 Pg C in the top 3 m of their soils^21–23^. The mobilization and conversion of just a fraction of this C pool into the greenhouse gases CO_2_ and CH_4_ and their release to the atmosphere could have strong positive feedbacks to global climate^23–25^. Modern terrigenous dissolved organic carbon (DOC) is an important component of Arctic C cycling^26–27^, but uncertainties remain regarding changes in the fluvial export of DOC in response to future climate and permafrost changes^20,28^. For example, differing forms of disturbance have been shown to result in either an increase^20,29–30^ or decrease^31,32^ in the concentrations and flux of DOC from permafrost-underlain watersheds. Limited studies also indicate close correlations between particulate organic carbon (POC) and suspended sediment (SS) erosion, noting increased POC in runoff from watersheds impacted by physical disturbance^33,34^. However, the flux and fate of POC in Arctic fluvial networks is the least known component of fluvial C cycling^19^. Quantifying the multiyear response of both DOC and POC export to differing forms of permafrost disturbance is crucial for understanding controls on the transfer of organic carbon (OC) from terrestrial surfaces to aquatic ecosystems in a changing Arctic climate^20,35–37^.

Climate models consistently project amplified Arctic warming^6^ together with increased summer rainfall^38–39^, which may change Arctic systems from a nival (snowmelt) to a pluvially (rainfall) dominated runoff and material transfer regime. Late summer rainfall events have the potential to mobilize considerable quantities of terrestrial material, as they couple hillslopes with stream networks when DOC, major ions, SS and POC are readily available for transport. Therefore, the projected increase in the magnitude and frequency of summer rainfall^38–39^ is likely to have substantial impacts on seasonal material flux from Arctic watersheds^40–41^. If terrestrial material from disturbed permafrost hillslopes are coupled with the fluvial system, this may have adverse effects on downstream aquatic ecosystems^9,42^. However, the nature of these shifts and the persistence of these cumulative impacts on fluvial material flux remain poorly understood, particularly in the High Arctic.

This study documents the response of fluvial fluxes to a decade of hydrometeorological change and resulting permafrost disturbance in two small headwater slope streams at the Cape Bounty Arctic Watershed Observatory (CBAWO) in the Canadian High Arctic (~ 75°N). We use a multiyear data set (2006–2017; excluding 2011, 2013 and 2015) integrating multiple variables, including major ions, SS, and OC to advance our understanding of how both thermal and physical permafrost disturbance alters all of the major components of fluvial flux. This integration includes analysis of the first multiyear record of fluvial DOC_flux_ and POC_flux_ from paired headwater slope streams affected by differing forms of permafrost disturbance from the Canadian High Arctic. Expanding on previous shorter-term studies (e.g., SS [2007–2012]^43^, major ion [2007–2014]^44^), this uniquely integrated data set highlights the relative importance of thermal disturbance and physical disturbance in determining the evolution of high-latitude biogeochemistry in response to shifting mobilization drivers.

## Field site

**Figure 1:**
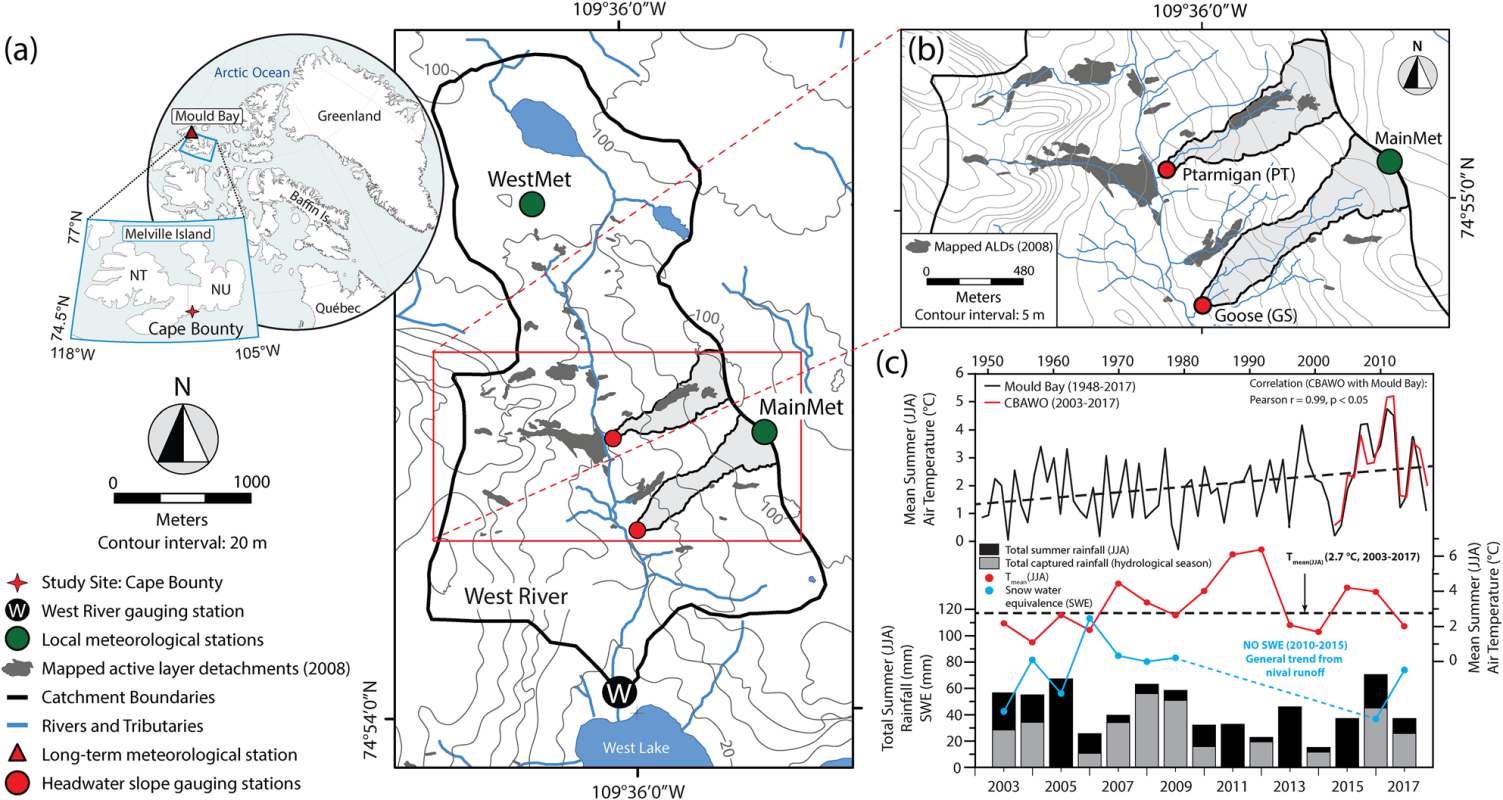
(**a**) Location of the Cape Bounty Arctic Watershed Observatory (CBAWO). (**b**) Study headwater slopes within the West river watershed. (**c**) Regional summer (June–August; JJA) surface air temperatures since 1948 (Mould Bay) (top), and summer surface air temperatures, total seasonal rainfall and watershed snow water equivalence (SWE) at the CBAWO, 2003–2017 (WestMet) (bottom). Total captured rainfall (grey) is the percentage of the total summer rainfall (black + grey) observed during the hydrological monitoring period each year. Watershed boundaries are shown, and the locations of the headwater-slope stream gauging stations and meteorological stations are indicated. Active layer detachments (ALDs; physical permafrost disturbances) that formed in 2007 are indicated as filled grey polygons and represent mapped extents as of August 1, 2008^74^. Basemap prepared in ArcGIS® (version 10.5) from NTS 1:50,000 map 78F/15 (UTM, WGS 84). Contains information licensed under the Open Government License—Canada.

This research was conducted at the CBAWO, Melville Island (NU) in the Canadian High Arctic (74°54′N, 109°35′W; Fig. 1a). The CBAWO is comprised of paired watersheds (West and East rivers, unofficial names), with instrumented small, headwater slope streams in the larger West river watershed. This site is underlain by continuous permafrost (~ 500 m thick) and characterized by a polar desert climate (MAAT: − 14.8 ± 1.3 °C) with limited annual precipitation and runoff (< 150 mm year^−1^)^41,45^. Mean summer (June–August; JJA) air temperature (MSAT_(JJA)_) at the CBAWO is 2.7 ± 1.4 °C (2003–2017) and the mean seasonal active layer thickness is between 0.7 and 1.0 m^45^. Extensive deposits of unconsolidated early Holocene marine and Late Glacial sediments drape the underlying sedimentary bedrock (folded Devonian sandstones and siltstones)^46^.

We focus on two small headwater slope streams within the West river watershed: Ptarmigan (PT; 0.21 km^2^) and Goose (GS; 0.18 km^2^) (Fig. 1b). PT represents a channelized thermally and physically disturbed watershed, while GS represents a physically undisturbed, but thermally disturbed watershed. Both watersheds in this study were altered by active layer thaw impacting the upper permafrost. Additionally, 10.8% of the areal extent of the PT watershed was physically disturbed by two localized active layer detachments (ALDs) at the end of the 2007 runoff season, after hydrological monitoring had ended. Maximum headwall erosion of the ALDs was ~ 45 m ± 5 m over a four-year period before stabilizing at the end of the 2011 season (Supplementary Fig. S1). Surface ponding is evident in several areas within the physical disturbance scar. Prior to 2007 there was no evidence for recent physical disturbance at the CBAWO^43^.

In PT, physically disturbed areas are coupled with the internal fluvial drainage network (Supplementary Fig. S1), transporting eroded material downslope to the larger West river channel. In GS, surface runoff occurs as diffuse flow over vegetated water tracks. The PT and GS watersheds experience broadly similar terrain characteristics and hydrological regimes. Soils are typical cryosols^47^, with a thin organic layer (< 5 cm) and low OC content (~ 1–3%) that developed from largely unaltered parent material^48^. The mean slope of the watersheds are low (PT: 3.9°; GS: 3.2°), and vegetation is characterized as prostrate dwarf-shrub tundra^49^ with a heterogeneous cover composed of wet, mesic, and polar desert communities^40^. Both streams have a seasonal flow regime (snow-limited) where channel runoff typically begins in early- to mid-June, with rapid flow cessation after ~ 7–12 days. In most years, runoff is rejuvenated in the late-season (July–August) by rainfall events of varying magnitude, with the intensity of pluvial runoff largely controlled by antecedent soil moisture conditions^50^.

## Materials and methods

Monitoring of headwater slope streams began prior to physical permafrost disturbances, providing pre- (2006–2007) and post-disturbance (2008–2017) records to evaluate their impact on dissolved and particulate fluvial fluxes. For coherency among records and to provide the finest temporal resolution across all years, we reprocessed all variables from raw data into daily means using a consistent methodology. We use propagation of uncertainty methods to: (1) account for the measurement error associated with instrumentation and analytical methods; and (2) determine how these uncertainties propagate through concentration and flux determinations, using the sum of quadrature method^51^. Herein, fluxes are defined as the total watershed export (kg; flux and export used interchangeably), and yield refers to watershed-normalized flux (kg km^−2^). We compare watersheds by normalizing flux by runoff (kg mm^−1^), since runoff is calculated as the watershed area-normalized sum of discharge (mm). Further, we compare different hydrological periods (snowmelt, baseflow, rainfall) and geomorphic change over time in terms of runoff-normalized flux. An increase (or decrease) in the flux per unit of runoff may indicate an increase (or decrease) in material export from watersheds. All values are reported as ± 1 standard deviation (± 1σ).

### Climatology

Climate data were obtained from local and regional meteorological stations proximal to the CBAWO. Local temperature and rainfall data were collected hourly from the WestMet meteorological station and barometric pressure data were collected at 10-min intervals at MainMet (Fig. 1a)^41^. Rainfall data from WestMet (2003–2017) were used to construct a 72-h intensity, duration, frequency (IDF) curve for the CBAWO (Gumbel EV1 distribution^52^) to estimate the recurrence interval for each rainfall event. Additional Environment and Climate Change Canada monthly climate data for Mould Bay, NT (~ 300 km west of the CBAWO, Fig. 1a inset), the nearest long-term (1948–2017) station, were utilized to evaluate longer-term annual hydrometeorological change^53^.

### Hydrology

Stream stations located at the outlet of watersheds, comprised of rectangular weirs (2006–2008) and 20 cm (8″) cutthroat flumes (2009–2017), were equipped with Onset U20 (± 0.5%, 2006–2014) and vented-Stevens SDX (± 0.3%, 2016–2017) pressure transducers and Onset H22 loggers to measure water level at 10-min intervals. Water level measurements collected using Onset U20 sensors were corrected for barometric pressure using Onset U20 (± 0.3%, 2007–2011) and CM50 (± 0.4%, 2011–2017) sensors at MainMet (Fig. 1a). Water level was converted to discharge (Q) using annual, site-specific stage-Q rating curves. Manual rating of stations were carried out with a Swoffer Instruments current meter (± 1%) periodically to confirm rating curves^43^. Mean Q uncertainty is calculated to be ± 4–6%. Seasonal hydrographs were partitioned into nival (snowmelt), baseflow (low flow, little-to-no diurnal variability), and pluvial (rainfall) hydrological periods based on subjective observed changes in the runoff regime^41,54^.

### Dissolved organic carbon and major ion

Concentrations of dissolved organic carbon ([DOC]) and major ions ([major ion]) were determined from surface water samples collected manually at: (i) approximately daily low and high Q (1000 h and 1700 h, respectively) during snowmelt and following rainfall; and (ii) once daily (1700 h) during low flow conditions until surface-runoff cessation. Water samples were collected in 1 L high-density polyethylene bottles, which were tripled rinsed with stream water before sample collection. Water samples for [DOC] were vacuum filtered through combusted (400 °C for > 4 h) 0.7-μm GF filters with a glass filtration apparatus within 4 h of collection^32^. Filtered samples were stored in 45 ml amber EPA vials with Teflon-lined septa and refrigerated in the dark (< 4 °C) following previously published methods^32,44^ until analysis using a Shimadzu TOC-VPCH/TNM system^32^. Filtered water samples were analyzed within 4–8 weeks of sample collection due to logistical limitations imposed by the remoteness of the CBAWO. We recognize that there was likely some DOC loss in our samples as a result of microbial processing during sample storage, but we are unable to quantify this effect. Further, we note that combusted 0.7-μm GF filters were not retained for POC analysis in this study (see Supplementary Information).

Water samples for [major ion] were vacuum filtered through sterile 0.22-μm polyvinylidene difluoride (PVDF) membrane filters with a polysulfone filtration apparatus in the field laboratory (within 4 h of collection), stored in 25 ml scintillation vials with no headspace, and refrigerated (< 4 °C) until analysis for [major ion] using a Dionex ICS 3,000 ion chromatograph^44^. Sample analyses were performed within 4–8 weeks of sample collection. The detection limits, calculated as three times the standard deviation of the lowest level standard, were less than 0.010 mg L^−1^ for most species, except for Ca^2+^ (0.053 mg L^−1^) and Mg^2+^ (0.022 mg L^−1^).[major ion] was calculated by summing all of the major anions and cations, with bicarbonate (HCO_3_^−^) concentrations estimated by charge balance^44^.

Daily fluxes (kg; DOC_flux_, major ion_flux_) were calculated as the product of total daily Q and daily mean [DOC] and [major ion] and summed for each season. Failure of refrigerators used to store water samples prior to analysis in 2009 and 2014 resulted in the loss of a significant number of DOC samples from PT. As a result, DOC_flux_ for PT in these years should be considered minimum estimates. Daily mean [major ion] in both watersheds for 2014 were estimated using the relationship between measured [major ion] and specific electrical conductivity (PT: r^2^ = 0.70, *n* = 13; GS: r^2^ = 0.82, *n* = 15). Average [DOC] uncertainty is calculated at ± 3–5% and ± 4–6% for [major ion]. Total uncertainties for seasonal DOC_flux_ and major ion_flux_ are estimated to be ± 6–10% and ± 5–9%, respectively.

### Suspended sediment and particulate organic carbon

Suspended sediment concentrations ([SS]) and POC concentrations ([POC]) were determined from surface water samples collected concurrently with DOC and major ions. All samples were volumetrically filtered through non-combusted, pre-weighed 1-μm glass fiber (GF) filters, folded to retain sediment on the filter and stored in individual polyethylene bags for [SS] and [POC] quantification. Although combusted 0.7-μm GF filters were not available for POC analysis in this study, extensive testing comparing non-combusted 1-μm GF filtration versus combusted 0.7-μm GF filtration showed negligible differences in [POC] from these watersheds at the CBAWO (≤ 1σ; see Supplementary Information), similar to observations from the Mackenzie River^55–56^.

In the laboratory, GF filters were freeze-dried prior to weighing and weighed twice to determine [SS]. Average [SS] uncertainty is ± 4% ([SS] > 10 mg L^−1^) and ± 10% ([SS] < 10 mg L^−1^)^43^. To determine POC content, filters were acid fumed with 50 ml of 6% trace metal grade sulfurous acid (H_2_SO_3_) for 20 h^57,58^ and oven-dried overnight at 50 °C. After fumigation, filters were sub-sampled into quarters, weighed individually, and pelletized in foil cups. Sample pellets were analyzed for organic carbon (OC) content using a LECO TruSpec CN elemental analyzer. Process blanks and LECO certified reference materials (LOT 1000: 10.8 ± 0.26% C, 0.86 ± 0.03% N) were run at the beginning and throughout every run to ensure consistency and determine instrument accuracy and stability. POC mass was calculated as the product of the sediment mass retained on the filter and the proportion of sediment composed of C (wt%), which was divided by filtered water volumes to determine [POC]. Average [POC] uncertainty was ± 6% ([POC] > 10 mg L^−1^) and ± 13% ([POC] ≤ 10 mg L^−1^). Daily fluxes (kg; SS_flux_, POC_flux_) were calculated with the product of total daily Q and daily mean [SS] and [POC] and summed for each season. Total uncertainties for seasonal SS_flux_ and POC_flux_ are estimated to be ± 4.5–10% and ± 8–15%, respectively.

### System response to disturbance

The double-mass curve (DMC) approach^59^ was used to identify changes in linear correlations (slope) between cumulative records of SS (POC is a component of SS), DOC, and major ion yield (kg km^−2^) plotted as individual functions of cumulative runoff (mm). In undisturbed watersheds, because runoff is the primary driver of material flux, cumulative plots between variables plot as an approximate linear relationship with unchanging slope^59^. Therefore, if a statistically significant change in slope between variables is present, we can identify changes to the system brought about by external perturbation (e.g., thermal and/or physical disturbance). Here, we use the DMC approach in combination with one-way analysis of covariance (ANCOVA) to identify significant changes in slope through the period of observation^41^.

## Results

### Observed hydrometeorological change (2003–2017)

Daily temperature records from the CBAWO show a statistically significant correlation with Mould Bay (2003–2017; r = 0.99, *p* < 0.05), providing a representative record for assessing longer-term climatological change^41^ (Fig. 1c). Longer-term regional MSAT_(JJA)_ showed a clear increase of ~ 2.0 °C between 1948–2017 at Mould Bay, with seven of the ten warmest summers on record occurring in the past decade. At the CBAWO, eight of the fifteen years of measurement have local MSAT_(JJA)_ above the longer-term mean (2.7 °C; 2003–2017) at the site. The timing of the ALDs (July 2007) was the warmest month since observations began at Mould Bay. In this study, we define thermal disturbances at two temporal scales: single-thaw season (JJA) thermal disturbances in the summers of 2007, 2012 and 2016 (the three warmest thaw seasons) and a period of multiyear thermal disturbance from 2007 to 2012 (a period of consistent above average summer air temperatures; Fig. 1c). We note summer 2011 as an additional single thaw season thermal disturbance, but no hydrological measurements are available to quantify any fluvial impact.

**Figure 2:**
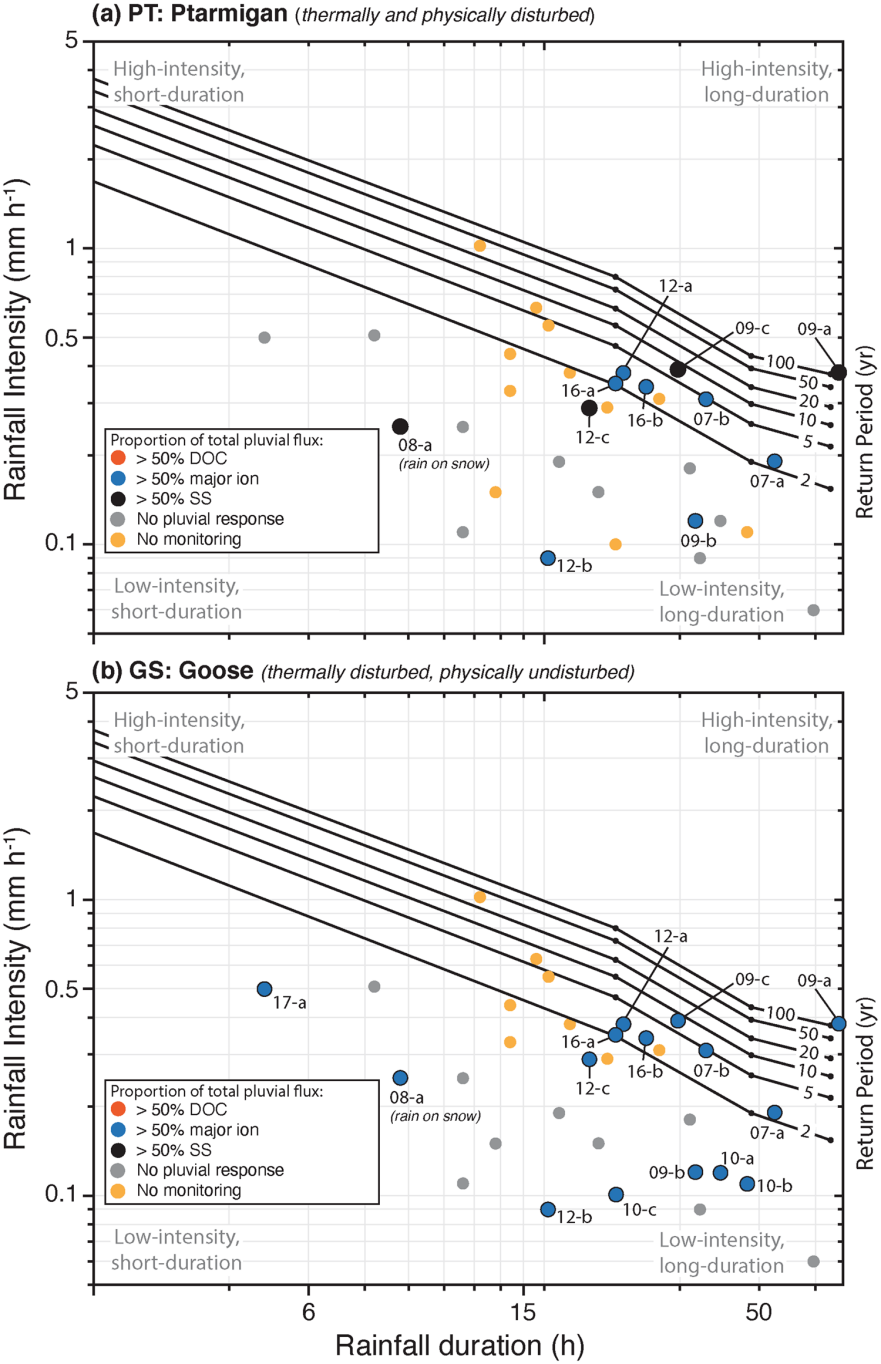
The 72-h intensity–duration–frequency (IDF) curve for the CBAWO. Overlain is the contribution of DOC_flux_ (> 50% total pluvial flux), major ion_flux_ (> 50% total pluvial flux) and SS_flux_ (> 50% total pluvial flux) plotted against rainfall intensity (mm h^−1^) and duration (h) for individual rainfall events with a measurable response. Note that not all rainfall events produce any measurable pluvial response at this watershed scale (grey dot). Due to logistical limitations, late-season (August) rainfall events are often not monitored (yellow dot). IDF graph was based on rainfall data (2003–2017) collected at the WestMet meteorological station^41^.

There was a general decline in watershed snow water equivalence (SWE) and subsequent nival runoff throughout the period of observation at the CBAWO^41^ (Fig. 1c). Shorter-term trends in SWE and total runoff showed an increase between 2007–2010 (~ 30 mm year^−1^) and a decreasing-trend thereafter (~ 10 mm year^−1^; Fig. 1c). Given the relatively short duration of available snow and rainfall records, evaluation of any changes in the longer-term trend of precipitation is beyond the scope of these data. Low-intensity, short-duration rainfall events were common (annual) at the CBAWO (~ 0.30 mm h^−1^, 24 h, < 2-year return period; Fig. 2). Major rainfall events (> 5-year return period or a 20% chance of exceedance in any given year) occurred less frequently over the period of observation (Fig. 2). Two-consecutive major rainfall events recorded in 2009 (within one week of each other) were estimated to have ~ 100-year (0.38 mm h^−1^; 75 h) and ~ 8-year (0.39 mm h^−1^; 33 h) return periods (1% and 12.5% chance of exceedance in any given year), respectively (09-a and 09-b; Fig. 2, Supplementary Table S1).

### Hydrology

**Figure 3:**
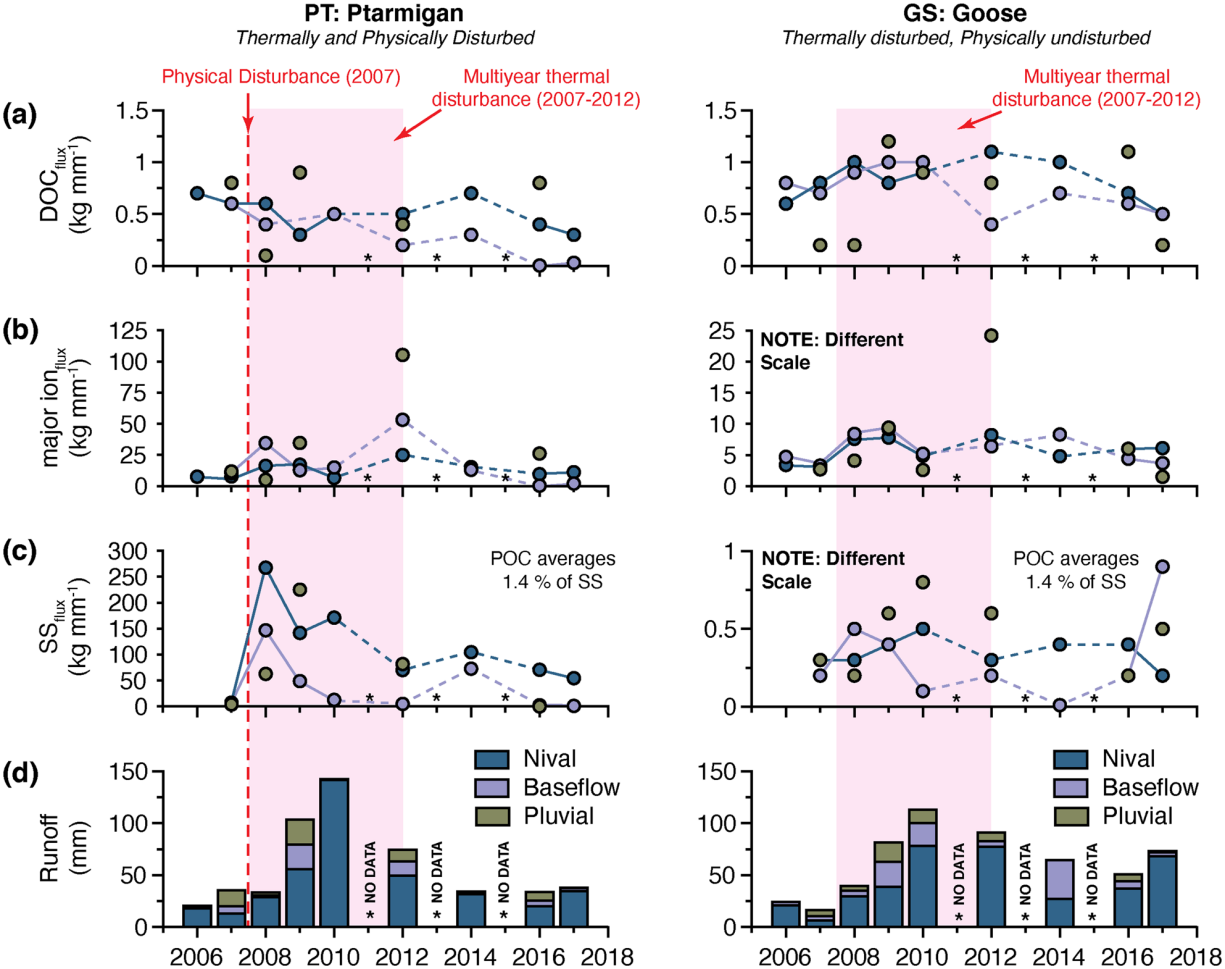
Runoff-normalized fluvial fluxes (kg mm^−1^) for all hydrological periods (nival, baseflow, pluvial) from Ptarmigan (PT) and Goose (GS), 2007–2017. (**a**) Dissolved organic carbon (DOC_flux_). (**b**) Major ions (major ion_flux_). (**c**) Suspended sediment (SS_flux_). Note that POC averages 1.4% of the SS in both watersheds and mirrored the patterns of SS_flux_ shown graphically here. (**d**) Annual runoff separated into hydrological periods. The timing of multiyear-thermal (2007–2012) and physical disturbances (2007) are shown.

Seasonal trends in discharge were similar in both streams. Most of the annual runoff occurred during the short nival period (44 ± 39 mm in PT and 43 ± 26 mm in GS) (Fig. 3d; Supplementary Table S2). Pluvial runoff averaged 10 ± 8 mm and 8 ± 6 mm in PT and GS, respectively (Fig. 3d). Low-intensity, short-duration rainfall events did not produce measurable hydrological responses for all recorded rainfall inputs (Fig. 2). Furthermore, late season rainfall events occurred most years after hydrological monitoring had ceased (Fig. 2). Baseflow hydrological periods averaged 7 ± 8 mm and 13 ± 12 mm in PT and GS, respectively (Fig. 3d).

### Controls on fluvial material transfer

Seasonal and interannual variability in fluvial material transfer was largely driven by variability in runoff. In general, the nival hydrological period accounted for the majority of fluvial fluxes in PT (≥ 70%), with an average nival SS_flux_ of 6,258 ± 7,811 kg (POC is 1.4% of SS), DOC_flux_ of 22 ± 20 kg, and major ion_flux_ of 539 ± 403 kg (Supplementary Table S2). In the GS watershed, the nival period accounted for an average ≥ 65% of annual SS_flux_ (16 ± 12 kg; POC is 1.4% of SS), DOC_flux_ (36 ± 26 kg), and major ion_flux_ (267 ± 192 kg).

Low energy baseflow runoff accounted for an average of ≤ 5% of the annual SS_flux_ in PT (213 ± 397 kg) and ~ 12% annual SS_flux_ in GS (3 ± 3 kg), < 5% of the annual DOC_flux_ (1 ± 1 kg) and < 10% of the annual major ion_flux_ (151 ± 251 kg) in PT, and an average of > 20% of both the DOC_flux_ (10 ± 11 kg) and major ion_flux_ (89 ± 106 kg) in GS (Supplementary Table S2). On average pluvial runoff transported ≤ 20% of the annual SS_flux_ (1,225 ± 2,101 kg) and ≥ 30% of the annual DOC_flux_ (8 ± 8 kg) and major ion_flux_ (414 ± 479 kg) in PT, and < 15% of all fluxes from GS (SS_flux_: 6 ± 6 kg; DOC_flux_: 9 ± 8 kg; major ion_flux_: 73 ± 78 kg.

Distinct pluvial responses were observed for rainfall events of varying magnitude during the period of instrumentation (*n* = 11 in PT and *n* = 15 in GS; Fig. 2). Plotting the proportion of the total pluvial flux (sum off all flux parameters; %) from both watersheds and associated rainfall events shows a distinct difference in the relationship between material flux and rainfall intensity and duration. Proportionally, pluvial runoff during lower magnitude rainfall events (< 2-year return period) transported more DOC and major ions than SS from the PT watershed (Fig. 2a; Supplementary Fig. S6a and Table S1). These data also indicated that the intensity and duration of rainfall events had to increase in magnitude (> 5 to 100-year return period event) to export proportionately more SS than DOC and major ions from PT (09-a and 09-c; Fig. 2a; Supplementary Fig. S6a and Table S1). An early season rain-on-snow event (08-a) and consecutive, multiple lower magnitude rainfall events (12-c) were proportionately SS dominated in PT (Fig. 2a). In contrast, the relationship between rainfall magnitude and the relative composition of pluvial material fluxes from GS were dominated by major ion_flux_ and DOC_flux_ (Fig. 2b; Supplementary Fig. S6b and Table S1). Pluvial events transported proportionately more SS in 2010 compared to all other years in GS (Supplementary Fig. S6b).

### Patterns of DOC and major ion transfer

Mean annual [DOC] and annual DOC_flux_ were consistently higher (on average ~ 2.5 times) in GS compared to PT (Supplementary Tables S2 and S4) but mean annual [major ion] and annual major ion_flux_ were consistently higher in PT compared to GS (Supplementary Table S2 and S4). In PT, [DOC] and [major ion] were only significantly correlated (*p* < 0.05) in 2007 (r = 0.53), 2009 (r = 0.38), 2012 (r = 0.40) and 2014 (r = 0.67) (Supplementary Table S5). In GS, [DOC] and [major ion] were moderately to strongly correlated in all years (r = 0.39–0.91, *p* < 0.05) and not significantly (*p* > 0.05) correlated in 2007, 2010 and 2017 (Supplementary Table S5).

Interannual changes in DOC export differed between the two watersheds (Fig. 3a). In GS, runoff-normalized DOC_flux_ (kg mm^−1^) during nival and baseflow hydrological periods increased from 2007 to 2012, and then declined modestly between 2014 and 2017 (Fig. 3a; Supplementary Table S3). In contrast, runoff-normalized DOC_flux_ during these hydrological periods steadily decreased during the period of observation in PT (Fig. 3a; Supplementary Table S3). In years with proportionately more pluvial runoff, pluvial events had equal or higher runoff-normalized DOC_flux_ compared to nival and baseflow runoff periods (Fig. 3). Interannual variability in mean [DOC] followed these same trends in both watersheds (Supplementary Table S4).

Multiyear changes in the major ion_flux_ were similar in both watersheds, with the greatest magnitude of change observed in PT. In both watersheds, runoff-normalized major ion_flux_ during nival and baseflow runoff increased nonmonotonically throughout the period of observation (Supplementary Table S3). Runoff-normalized major ion_flux_ as a result of pluvial runoff similarly increased during this time (Fig. 3b; Supplementary Table S3). Runoff-normalized major ion_flux_ during all hydrological periods were highest in 2012 in PT (warmest JJA on record), but only substantially higher during pluvial runoff in GS in the same year (Fig. 3b). Concentrations and flux of major ions remained elevated from 2014–2017 (relative to 2006–2007) in both watersheds (Fig. 3b; Supplementary Tables S2 & S4).

### Patterns of SS and POC transfer

[SS] and [POC] were strongly linearly correlated (r^2^ = 0.96–0.99, *p* < 0.05), co-varying each year in both streams (Supplementary Fig. S7).[SS] and [POC] were on average 200 times greater in PT compared to GS (Supplementary Table S4). The proportion of POC in SS ranged from 0.9 to 2.1%, with POC averaging 1.4% of the SS in both watersheds (Supplementary Table S4). The slopes of the correlation between [SS] and [POC] varied between years suggesting the need for annual, site-specific relationships to be established (Supplementary Fig. S7).

Interannual changes in SS differed between watersheds due to the type of permafrost disturbance (Fig. 3c). Runoff-normalized SS_flux_ in PT were low prior to the 2007 physical disturbance event (Fig. 3c; Supplementary Table S3). In the year immediately the following physical disturbance (2008), runoff-normalized SS_flux_ during all hydrological periods were 15–30 times greater relative to 2007 (Fig. 3c). SS_flux_ continued to increase in PT until the end of 2010 and then rapidly declined between 2012–2017 but remained elevated relative to pre-disturbance flux (Fig. 3c). Runoff-normalized SS_flux_ during pluvial events followed similar trends through time (Fig. 3c), and runoff-normalized POC_flux_ from the PT watershed mirrored the changes in SS_flux_ (Supplementary Table S3). In contrast, runoff-normalized SS_flux_ (and therefore POC_flux_) in GS varied primarily with annual runoff (Fig. 3c; Supplementary Table S3).

Pearson linear correlations between seasonal [SS] and [DOC] were similar between watersheds (Supplementary Table S5). In both watersheds, [SS] and [DOC] were not significantly correlated (*p* > 0.05) in all years except during 2008 in PT (r = − 0.55, *p* < 0.05) and 2010 (r = 0.47, *p* < 0.05) and 2017 in GS (r = 0.57, *p* < 0.05).

### Impact and response to disturbance

**Figure 4:**
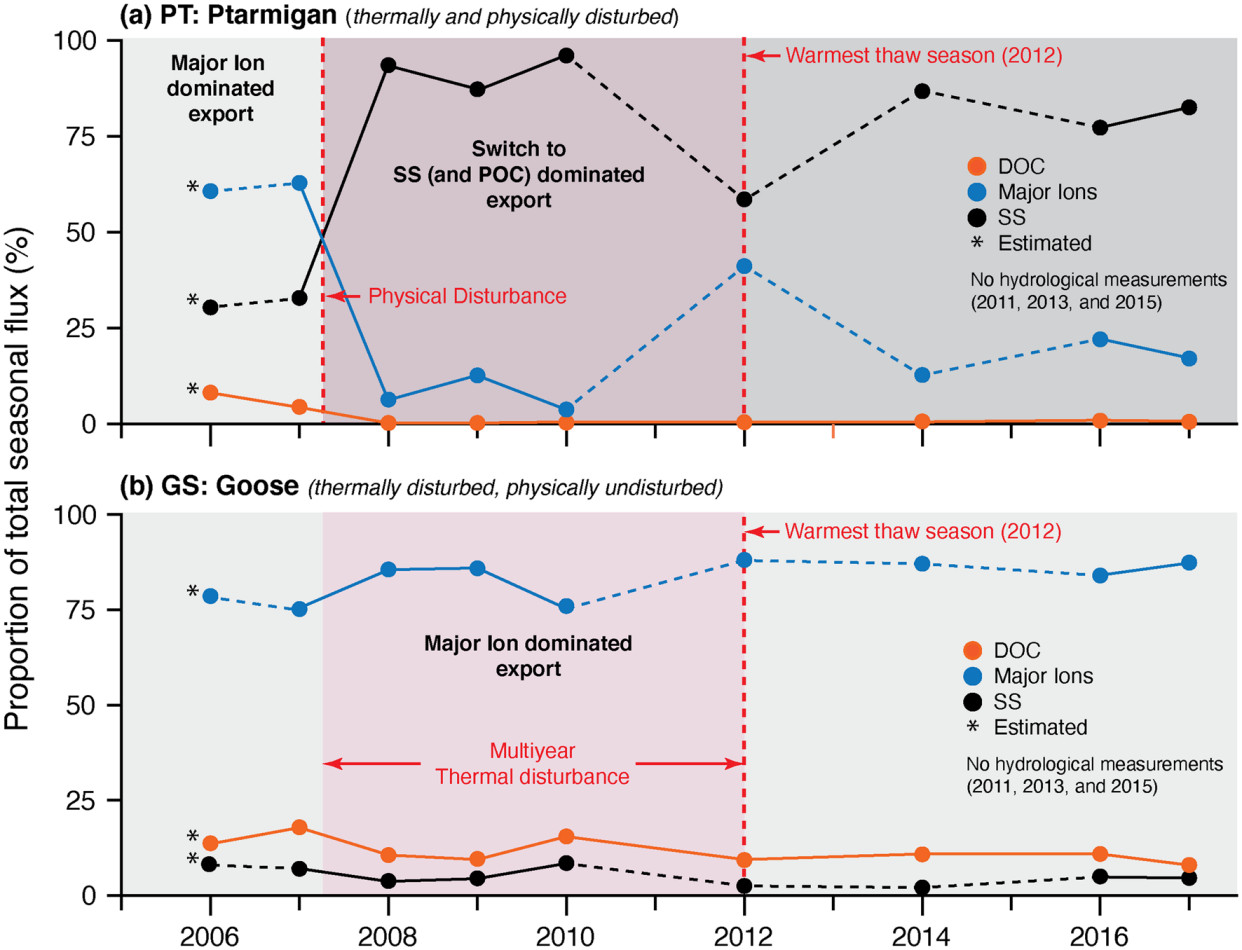
Relative contributions of DOC_flux_, major ion_flux_ and SS_flux_ to the annual total flux (sum off all parameters) for (**a**) Ptarmigan (PT) and (**b**) Goose (GS). The timing of multiyear-thermal (2007–2012) and physical disturbance (2007) are shown. Note the transition from a dissolved to a particulate dominated system in PT immediately following thermal and physical disturbance. Estimates for the % of SS in 2006 were based on consistent longer-term averages in GS (2006–2017) and by estimating the SS_flux_ using pre-disturbance (2007) runoff-normalized SS_flux_ for the different hydrological periods in PT and applying them to 2006.

To further explore system response to disturbance, we compared the relative contributions of DOC_flux_, major ion_flux_, and SS_flux_ to the total annual fluvial flux (sum of all flux parameters; Fig. 4). Prior to disturbance (2006–2007) annual fluvial fluxes from PT were dominated by major ions (~ 60%) (Fig. 4a). We then observed a transition in the system (2008–2010) from a major ion to a SS dominated annual flux (~ 80–90%). During the 2012 thaw season we observed an increase in major ion_flux_ from both watersheds, with the most pronounced change in the seasonal proportionality observed in PT (Fig. 4a). In contrast to PT, major ion_flux_ and DOC_flux_ from GS dominated the annual flux in all years (~ 10% and ~ 80%, respectively; Fig. 4b).

**Figure 5:**
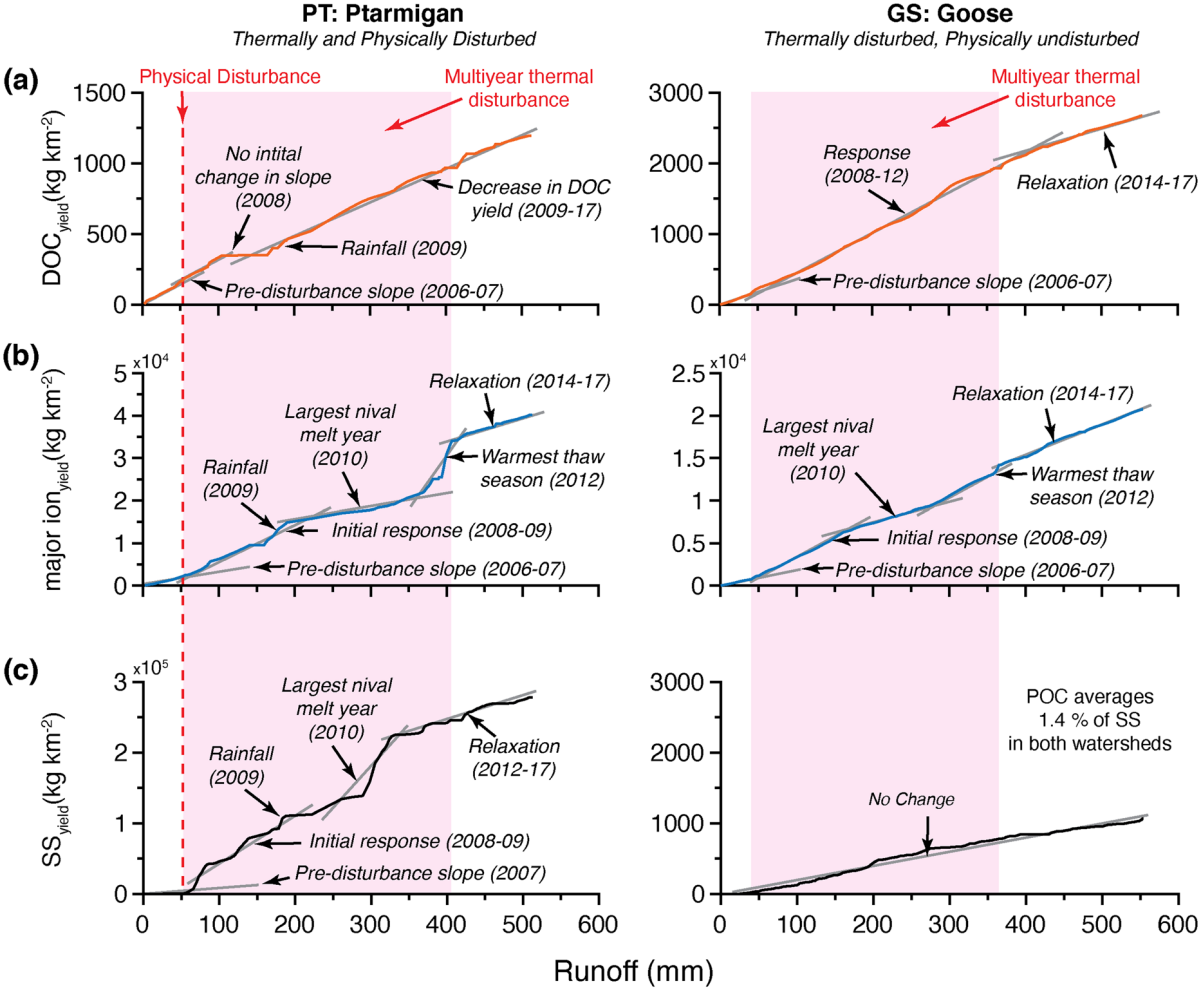
Cumulative (**a**) DOC_yield_ (kg km^−2^), (**b**) major ion_yield_ (kg km^−2^) and (**c**) SS_yield_ (kg km^−2^) plotted as individual functions of cumulative runoff (mm) for Ptarmigan (PT) and Goose (GS). Here we use the double-mass curve approach to identify statistically significant changes in slope (ANCOVA, *p* < 0.05), representing changes in the environmental conditions of a watershed. Major geomorphic events and hydrometeorological drivers are labelled.

Figure 5 shows cumulative yields (kg km^−2^) of DOC, major ions, and SS plotted as individual functions of cumulative runoff (mm) for both watersheds. In PT, DOC_yield_ showed no initial response to permafrost disturbance in 2008 and then the slope modestly declined between 2009–2017 (Fig. 5a). Immediately following physical disturbance in PT, SS_yield_ and major ion_yield_ increased (relative to pre-disturbance) before both records show a step-wise change in yield preceding the relaxation slope (Fig. 5b, c). The step-wise change in the SS_yield_ was in response to a period of major channel incision in PT during the largest nival melt year on record (2010; Fig. 5c). The step-wise change in major ion_yield_ was in response to the warmest thaw season on record with associated active layer deepening and significant pluvial runoff (2012; Fig. 5b), though DOC_yield_ showed no step-wise response in the same year (Fig. 5a). Major rainfall events in 2009 caused further step-wise change in all records in PT (Fig. 5). One-way ANCOVA showed that the slopes of observed changes in the cumulative relationships in PT are significantly different from each other (*p* < 0.05; Supplementary Table S6).

The slope of cumulative DOC_yield_ increased between 2008–2012, then declined modestly from 2014–2017 in GS (Fig. 5a). Changes in the relationship between major ion_yield_ and runoff in GS were similar to those observed in PT (Fig. 5b), but the magnitude of impact was less. For example, the step-wise increase in major ion_yield_ in 2012 was ~ 7 times greater in PT compared to GS (Fig. 5b). The relationship between cumulative SS_yield_ and runoff in GS represents a notionally ideal, physically undisturbed watershed with a near constant proportionality between variables due to the absence of geomorphic change leading to increased sediment availability and mobilization (i.e., no significant change) (Fig. 5c; Supplementary Table S6).

## Discussion and conclusions

Climate-driven thermal perturbations and the resulting physical disturbance of permafrost landscapes have multiple longer-term (≤ 10 years) effects on DOC, major ion, SS, and POC flux from small High Arctic headwater slope streams (~ 0.2 km^2^). Even though a decade-long dataset can be considered short-term, these data are the most complete multiyear pre- and post-disturbance record of its kind in the High Arctic and as a result, have shown previously unrecognized patterns of fluvial flux response to thermal and physical perturbation. Thermal perturbation predominately affects DOC and major ion flux, whereas physical disturbance is localized, but significantly alters all fluvial flux (DOC, major ion, POC, SS).

Multiyear thermal disturbance increased the seasonal flux of DOC from a small thermally disturbed, but physically undisturbed watershed at the CBAWO (Fig. 5a). In this watershed, runoff-normalized DOC_flux_ increased during the period of multiyear thermal disturbance (2007–2012), and then declined to background (2006–2007) flux by the end of the data record (Fig. 3a). It is unclear whether this increase in the flux of DOC was primarily caused by vegetation responding to warming temperatures (e.g., increased rates of photosynthesis and C incorporation into the ecosystem)^26–27^, increased microbial processing of SOM enabling DOM liberation with increasing temperature^19^, permafrost thaw and/or active layer deepening^20^, or the cumulative impact of these processes. Previous studies from the CBAWO showed that DOC_flux_ from this watershed was predominately sourced from modern plant-derived material and SOM^32^, and a single POC sample from the outlet of the watershed yielded a ^14^C age and δ^13^C value (− 29.1 ‰) indicative of more modern terrigenous-origin C^33^. However, significant (*p* < 0.05) correlations between [DOC] and [major ion] in most years suggest that increased DOC export may have partially been the result of permafrost thaw and/or active layer deepening (Supplementary Table S5). Increased multiyear fluvial DOC_flux_ in this system is likely explained by the cumulative impact of the increased availability and export of DOC leachate sourced from surrounding modern-vegetation and SOM as a result of warmer temperatures, and permafrost thaw and/or active layer deepening resulting from thermal perturbation^19,20,26,27,61–63^.

Localized physical disturbance decreased multiyear DOC export from a small thermally and physically disturbed watershed at the CBAWO (Fig. 5a). [DOC] and DOC_yield_ steadily declined following the formation of hydrologically-connected ALDs (Fig. 5a; Supplementary Tables S3–S4). The observed decline in DOC export was likely driven by: (1) the development of internal channels within newly exposed low OC mineral soils of the scar zone; (2) poor-hydrological connectivity through displaced SOM at the toe of ALDs (Supplementary Fig. S1); and (3) enhanced microbial respiration (and mineralization of DOC to CO_2_) within physically disturbed watersheds^64–66^. Preferential sorption^67^ of available DOC to newly exposed mineral soils (SS) post-disturbance^31^ may also play a role in this system. The overall impact of physical permafrost disturbances on the flux of Arctic DOC is unclear; some studies also report decreases in the concentration and flux of DOC in disturbed watersheds^31,32^ while others have reported increased DOC_flux_ from various forms of physical disturbance^20,29–30^.

Localized physical disturbances lead to a fundamental shift in the primary export of C from small, headwater slope streams; from a DOC to a POC dominated fluvial system (Fig. 4a; Supplementary Tables S2–S4). Runoff-normalized SS_flux_ and POC_flux_ increased by an order of magnitude immediately following the formation of ALDs, but concentrations and fluxes rapidly declined within five years (Figs. 3c, 5c). SS_flux_ and POC_flux_ remained elevated relative to pre-disturbance a decade after the initial physical disturbance (Fig. 3c; Supplementary Tables S2–S3) suggesting that these systems are either: (a) continuing to recover back to a prior equilibrium state; or (b) have quasi-stabilized to a new, higher equilibrium^68^. Complimentary research at the CBAWO showed that surface runoff in PT exported permafrost-derived POC (6,600–6,740 year BP) to the downstream ecosystem^33^, and that the SOM was relatively labile and easily degradable^32,65–66^. The release of older, relatively labile POC as a result of physical disturbance (combined with increased DOC_flux_ during multiyear thermal disturbance) may stimulate enhanced biological productivity in both terrestrial and downstream aquatic ecosystems, increasing the release of greenhouse gases (e.g., CO_2_, CH_4_) to the atmosphere^22,24,60,69–71^ and enhancing a positive feedback on climate^23,70–71^. The release of older C due to permafrost disturbance has been observed across much of the Arctic^30,34–37,72^. These studies, coupled with our findings here, suggest an increase in the accessibility to and transfer of permafrost-derived terrestrial POC into downstream aquatic ecosystems in the next century as the Arctic continues to rapidly change^34,73^.

Thermal disturbance of the active layer increased the concentration and flux of major ions, irrespective of physical disturbance. Using the DMC approach^59^ we observed a similar pattern of major ion response in both watersheds, but the magnitude of impact was approximately an order of magnitude greater in the thermally and physically disturbed stream (Figs. 3b, 5b). This shows that the thermal response is more substantial when enabled by a physical disturbance. This is likely the result of physical disturbances removing overlying active layer soils and exposing transitional permafrost near the thaw boundary, bringing solute rich ice and soils to the surface where they are readily flushed into surface waters^15^. At the CBAWO, thermal and physical disturbances were shown to alter downstream major ion_flux_ in the larger river systems and have substantial downstream cumulative effects in freshwater lakes^40,42^, similar to findings from other Arctic watersheds impacted by permafrost disturbance^9–11,14,16–17^. Coupled climate-terrestrial models consistently predict widespread thermal and physical disturbance of the upper permafrost over the next century^7^, which is likely to increase the transfer of permafrost-derived major ions across the terrestrial-aquatic interface, altering the chemical composition of downstream aquatic ecosystems^42^.

Short-lived nival runoff (June) remained the primary hydrological driver in these systems, but the importance of pluvial runoff as driver of fluvial flux increased following permafrost disturbance. In both physically disturbed and undisturbed watersheds, pluvial runoff was more effective at transporting all flux parameters compared to nival runoff in almost all years (Fig. 3, Supplementary Table S2). In physically disturbed watersheds, the intensity and duration of rainfall events had to significantly increase in magnitude (e.g., 2009; > 5 to 100-year return periods) to transfer disproportionate quantities of SS by pluvial runoff (Supplementary Table S2). At the watershed-scale, these physical disturbances did not increase the multiyear downstream SS_flux_ at the CBAWO beyond an immediate short-lived pulse during baseflow in 2007^41^. However, pluvial runoff in response to these two high magnitude rainfall events in 2009 were the primary mechanism to overcome critical thresholds of additional sediment mobilization and transmit the signal of these physical disturbances downstream^41^. This suggests that particulate material transfer along the High Arctic fluvial sediment cascade is energy-limited under current hydroclimatic conditions (e.g., during all hydrological periods). However, global climate models consistently predict decreases in winter precipitation and increased moisture availability under warmer climates^6^, which is likely to increase the frequency and magnitude of summer rainfall^38–39^. If this occurs, the transition from a nival-dominated fluvial regime to a regime where rainfall runoff is proportionately more important will be the likely mechanism to overcome thresholds of additional sediment transfer in these environments. Simply put, physical disturbances may geomorphologically prime terrestrial surfaces for enhanced landscape erosion if future rainfall magnitudes exceed the current buffering capacity of hillslopes and the fluvial sediment cascade^41^.

This work constrains how different types of permafrost disturbance alter the composition and magnitudes of fluvial fluxes in discernible ways that advance our understanding of how to predict changes to biogeochemical cycling and aquatic systems. These changes are important to characterize because of the rapid nature of these shifts and the persistence of these impacts a decade after initial disturbance. Projected increases in the magnitude and frequency of physical disturbance will have profound impacts on lateral C fluxes across the terrestrial-aquatic interface, increasing the fluvial export of older permafrost-influenced POC. With recent and projected increases in temperature and changes to precipitation patterns, our results highlight the overall vulnerability of the Arctic to permafrost change, and its potential downstream impacts.

## Supplementary information

Supplementary Information 1.

Supplementary Information 2.

Supplementary Information 3.

Supplementary Information 4.
