# Supplementary material for: Differential impact of thermal and physical permafrost disturbances on High Arctic dissolved and particulate fluvial fluxes
Source: Sci Rep. 2020 Jul 16;10:11836. doi: 10.1038/s41598-020-68824-3 (PMC7366920; doi:10.1038/s41598-020-68824-3)
Supplement: Supplementary file 1 — Supplementary Information 1. [file 41598_2020_68824_MOESM1_ESM.pdf]

Supplementary Information for

Differential impact of thermal and physical permafrost disturbances on High Arctic dissolved and particulate fluvial fluxes

Beel, C.R.[1,2], Lamoureux, S.F.[1], Orwin, J.F.[1,3], Pope, M.A.[1], Lafrenière, M.J.[1], Scott, N.A.[1]

[1]Department of Geography and Planning, Queen's University, Kingston, ON K7L 3N6 Canada

[2]Yellowknife Research Office, Wilfrid Laurier University, Yellowknife, NT X1A 2P8, Canada

[3]Resource Stewardship Division, Alberta Environment and Parks, Government of Alberta, Calgary, AB T2L 2K8, Canada

Contents of this file:

1. Geomorphic evolution of active layer detachments (Fig. S1)
2. Particulate organic carbon (POC) method
    2.1. Blank corrections (Fig. S2)
    2.2. Duplicate samples (Fig. S3-S4)
    2.3. Replicate samples (Fig. S5)
3. Supporting Figures (Fig. S6-S7)
4. Supporting Tables (Tables S1-S6)

Data availability:

1. Climate: air temperature and rainfall for the CBAWO (2003-2017) available on Polar Data Catalogue (https://www.polardata.ca/).
2. Hydrology: discharge, suspended sediment, particulate organic carbon, dissolved organic carbon and major ion are available as supplementary files.
3. Supporting Tables S1-S4 are available as spreadsheets with the supplementary files.

1. Geomorphic evolution of localized active layer detachments

Headwall expansion and stabilization (2007-2011) of two localized active layer detachments (ALDs) in the thermally and physically disturbed Ptarmigan watershed (PT; Fig. 1a). Rapid headwall erosion followed the initial formation of ALDs (2007-2009), before slowing significantly as the ALD stabilized (Fig. S1c). Maximum headwall erosion is estimated between 35-50 ± 5 m over a five-year period (mean headwall erosion rate between 8.5-12.5 ± 5.0 m y^{-1}). ALDs developed new internal channels in 2008 and continued to incise 0.5-1.2 m deep until 2012[1]. Newly formed channels within the scar/track zone of the ALDs are well-connected with the preexisting downstream channel, readily transporting newly exposed terrestrial material downstream to the larger West river (Fig. S1c). The primary channel was diverted around the displaced active layer soils in the toe/compression zone (Fig. S1b), and surface runoff through the intact active layer is limited due to poor hydrological connectivity. Surface ponding is evident in several areas within the physical disturbance scar, and act to further disrupt the fluvial sediment cascade.

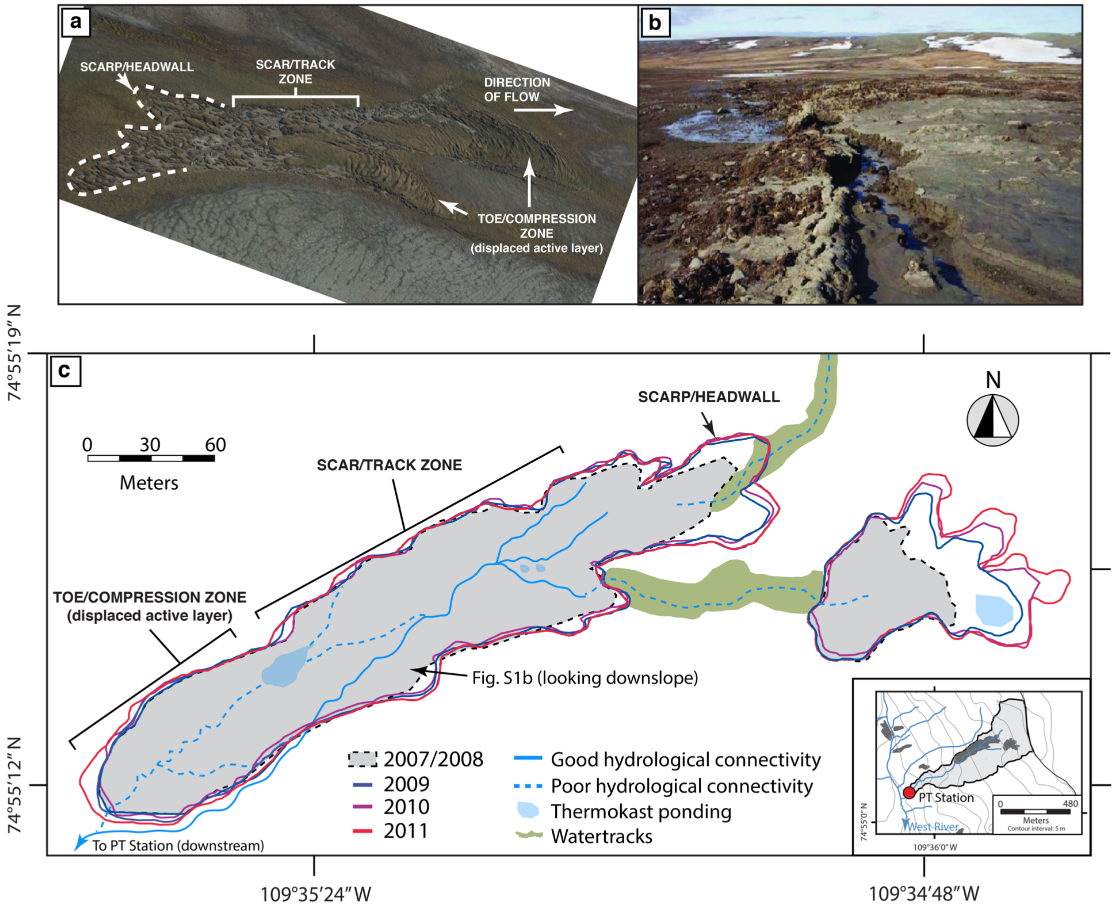

Fig. S1. (a) Annotated-oblique aerial photograph of a localized active layer detachment (ALD) at the CBAWO. (b) Photograph of the newly formed, well-connected channels within the scar/track zone of ALDs in PT (photo location indicated in (c)). Note that surface runoff has been redirected around the intact-active layer soils in the toe/compression zone. (c) Geomorphic evolution of localized ALDs in the PT watershed. Mapping was carried out annually with a handheld Garmin GPS. Hydrological connectivity of the internal channel configuration is indicated as mapped with handheld GPS in 2010. Photographs (a/b): S.F. Lamoureux.

2. Particulate organic carbon (POC) method

2.1 Blank corrections

It is important to note a key difference in our method of POC determination. Here, we opportunistically use the non-combusted, 1-µm glass fiber (GF) filters from suspended sediment concentration ([SS]) determination, which differs from standard methods that use combusted 0.7-µm GF filters. Although pre-combustion of filters is recommended to eliminate any initial C and to reduce blank values, combusted 0.7-µm GF filters were not available for POC analysis in this study. All combusted filters used for DOC sampling were discarded immediately following filtration in the field. 1-µm GF filters used in this study were manufactured by Whatman®, which are free of organic binders and typically have low C blank values[2]. We determined the OC content of 25 new 1-µm GF filters to determine a blank correction value to account for the lack of combustion. The average OC content of the blank filters was 0.001 ± 0.0004 % C (Fig. S2).

Process blanks and LECO certified reference materials (LOT 1000: 10.8 ± 0.26 % C, 0.86 ± 0.03 % N) were run at the beginning and throughout every run to ensure consistency and to determine instrument accuracy and stability. Blank values for LECO foil cups (n = 200) had an average OC content of 0.033 ± 0.006 % C (Fig. S2). All samples were corrected for these trace amounts of OC in blank filters and foil cups.

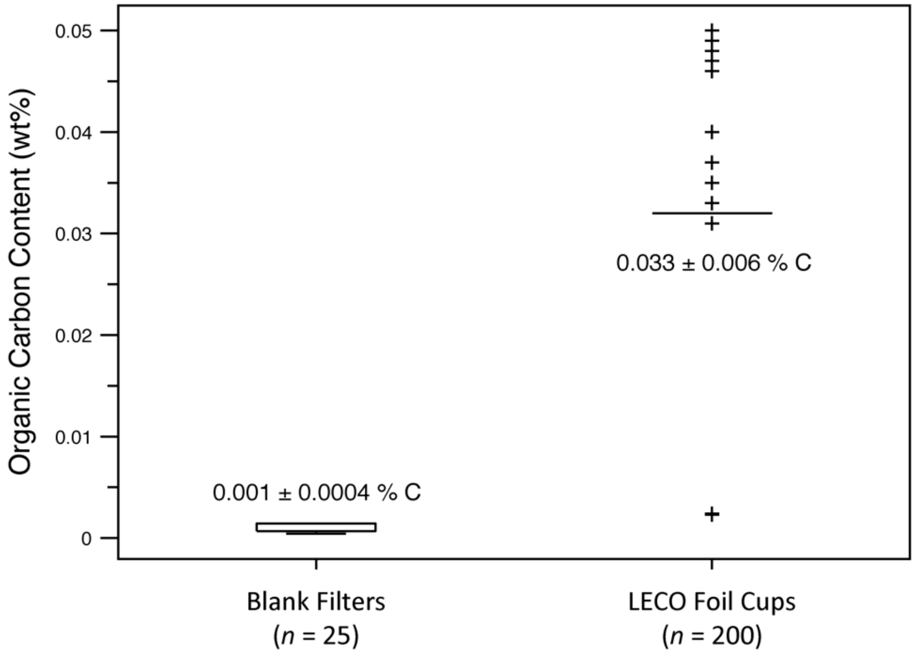

Fig. S2. Boxplot of OC content (wt%) in new 1-µm glass fiber filters and LECO foil cups. All blank filters were acid fumed following standard methods prior to analysis. All samples were corrected for these trace amounts of OC.

2.2 Duplicate Sampling

Duplicate water samples (n = 46) collected from four river systems of varying watershed size (10-100 km^2) at the CBAWO, during different hydrological periods (nival, baseflow, stormflow) in 2017 were used to compare differences between the [POC] measured from non-combusted 1-µm GF filters and combusted 0.7-µm GF filters. Studies of [POC] in the Mackenzie River noted that using 1.2-µm pore size GF filters versus filters with smaller pore sizes showed negligible differences in [POC][3-4]. Although the relationship is not 1:1, measured [POC] is strongly correlated (r^2 = 0.93) and within 1-standard deviation (± 1σ) using 1-µm and 0.7-µm GF filters (Fig. S3). Further, small root-mean-square-error (RMSE) relative to the linear correlation suggests little variation in [POC] between filters. Together, these findings suggest that the use of non-combusted, 1-µm GF filters provide an accurate measure of [POC] in this setting. We also compare the correlation between [SS] and [POC] for the different filters (Fig. S4). One-way ANCOVA indicates that the slopes are significantly different from each other (1-µm vs. 0.7-µm), but are within ± 1σ, further validating our use of filters in this study.

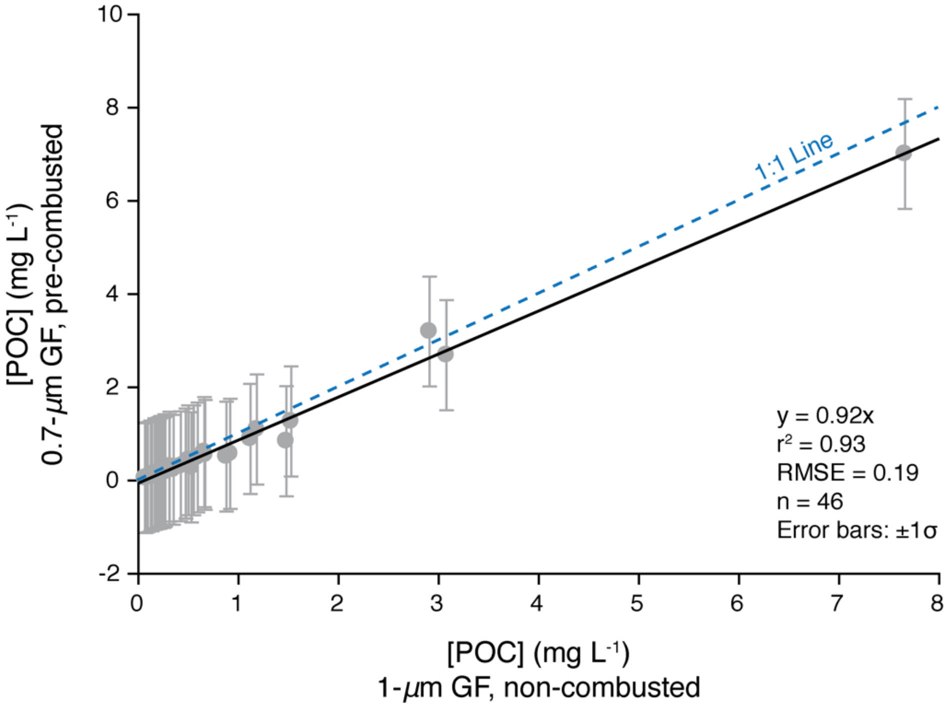

Fig. S3. Comparison of [POC] determined from duplicate water samples filtered through non-combusted 1-µm GF filters and combusted 0.7-µm GF filter. Duplicate samples were collected across the different hydrological periods (nival, baseflow, stormflow) from four river systems of differing catchment size (10-100 km^2) at the CBAWO. Although the relationship is not 1:1, measured [POC] are strongly correlated and within 1 standard deviation (± 1σ) of each other, with little variation around the 1:1 line (RMSE = 0.19).

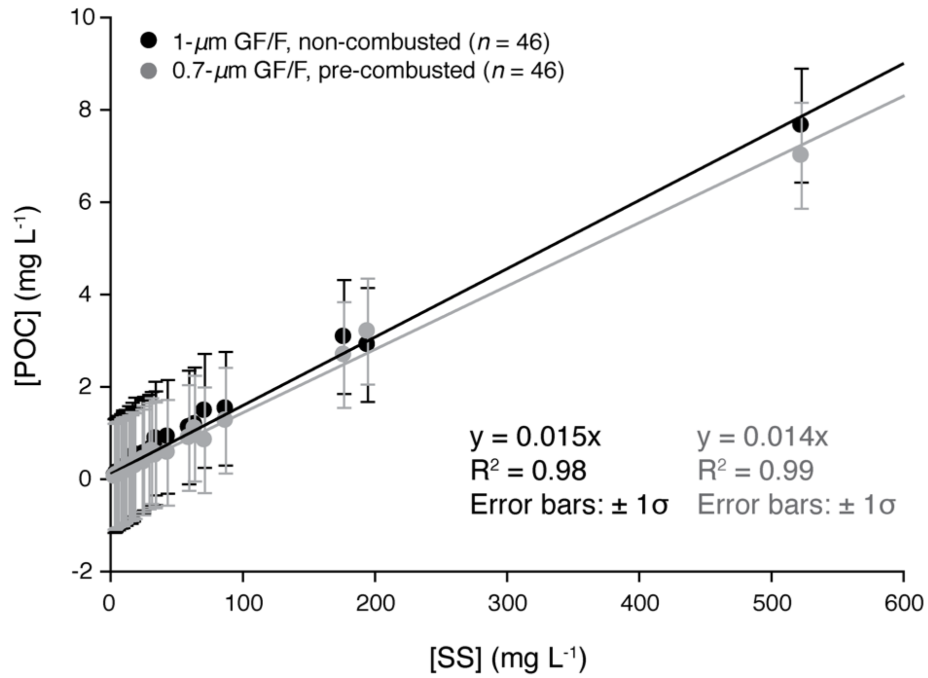

Fig. S4. Comparison of the relationship between [SS] and [POC] for duplicate water samples filtered through non-combusted 1-µm GF filters and combusted 0.7-µm GF filters. One-way ANCOVA indicates that the slopes are significantly different from each other, but within 1 standard deviation (± 1σ).

2.3 Replicate Samples

Due to subsampling of the filters into quarters for analysis (maximum size that for the LECO), randomly selected duplicate samples from both streams (*n* = 32 each for PT and GS) were analyzed (Fig. S5). Replicate samples are strongly correlated with each other ($r^2 = 0.99$, $p < 0.05$), have small RMSE relative to their respective linear correlations (PT: 0.5; GS: 0.04), and sample means equal to each other ($p < 0.05$), indicating that sub-sampling of the filters does not skew or bias the measured [POC] in both streams.

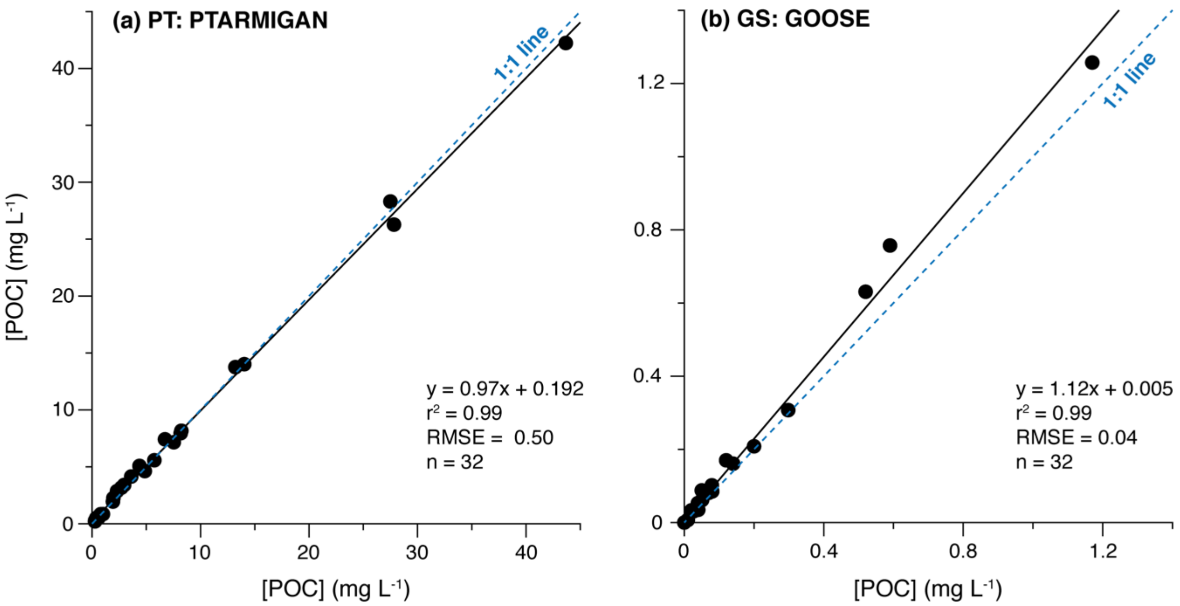

Fig. S5. Fitted linear relationships between [POC] for replicate samples for the (a) PT: Ptarmigan and (b) GS: Goose. Two-tailed t-tests indicate that the sample means are equal between both runs ($p < 0.05$). 1:1 line shown for reference (blue-dashed)

3. Supporting Figures

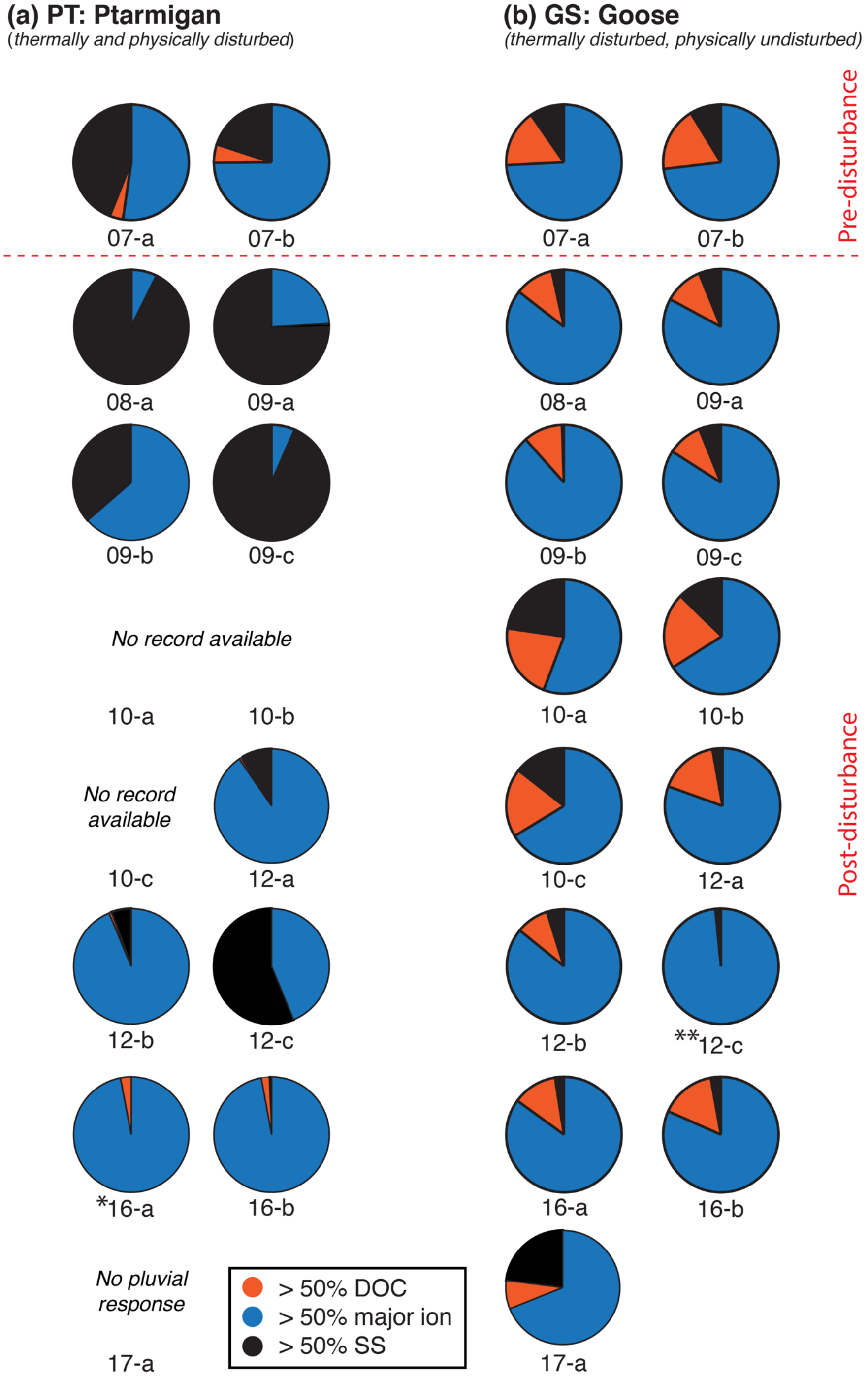

Fig. S6. Proportion of total pluvial flux (sum of all flux) for individual rainfall runoff responses in (a) Ptarmigan (PT) and (b) Goose (GS), 2007-2017. Simplified and displayed graphically in Fig. 2 as a single color denoting the dominance (>50 % of the total pluvial flux) of either DOC_{flux} (orange), major ion_{flux} (blue) or SS_{flux} (black). Data available in Supplementary Table S1.

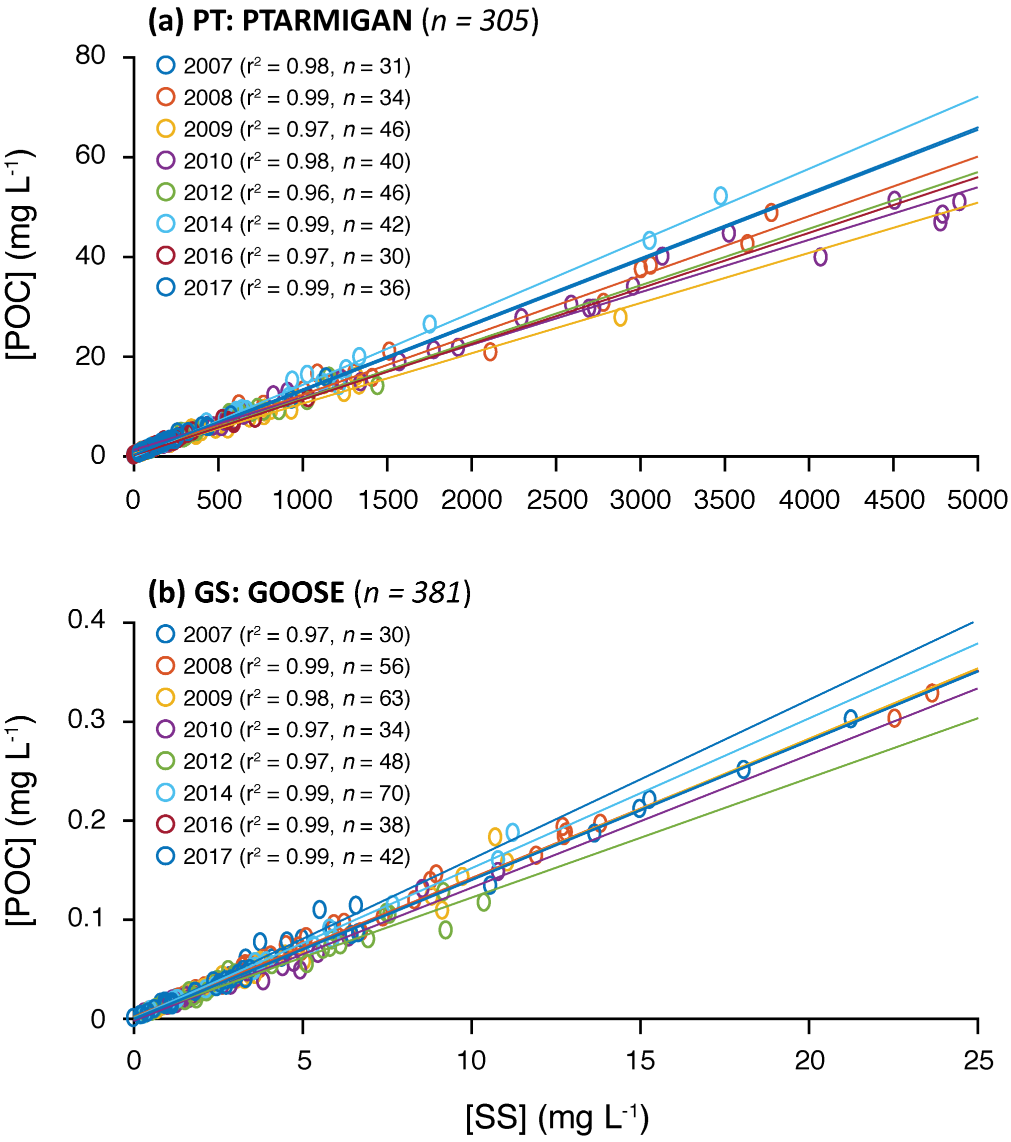

Fig. S7. Seasonal correlations between suspended sediment concentration ([SS]) and particulate organic carbon concentration ([POC]) for (a) PT: Ptarmigan and (b) GS: Goose, 2007-2017 (excluding 2011, 2013, 2015). Note that the range of [POC]/[SS] observed in the undisturbed GS are a fraction of that observed in the physically disturbed PT.

4. Supporting Tables

Table S1. Rainfall events with measurable pluvial responses for Ptarmigan (PT, *n* = 11) and Goose (GS, *n* = 15). Rainfall duration, intensity and estimated recurrence interval (years) are shown, as is the total pluvial runoff for each rainfall event, SS_{flux} (POC_{flux} is 1.4 % of SS), major ion_{flux}, and DOC_{flux} (kg). Seasonal timing, early (Jun), Mid (Jul), Late (Aug) indicated. Labels refer to the events displayed graphically in Fig. 2.

Site	Year	Label (Fig. 2)	Date	Season Timing	Rainfall Duration (h)	Rainfall Intensity (mm h^{-1})	Recurrence Interval (y)	Pluvial Runoff (mm)	SS_{flux} (kg)	major ion_{flux} (kg)	DOC_{flux} (kg)
PT	2007	07-a	22 Jun	Early	14	0.33	2	3.7	22.1	26.3	1.9
	2007	07-b	30 Jun	Early	54	0.19	5	11.6	40.5	152.0	10.6
	2008	08-a	24 Jun	Early	8	0.25	< 2	3.2	956.3	76.1	1.6
	2009	09-a	17 Jul	Mid	75	0.38	100	12.3	1474.9	470.2	10.2
	2009	09-b	24 Jul	Mid	36	0.12	< 2	1.9	50.4	88.2	*
	2009	09-c	26 Jul	Mid	33	0.39	8	9.8	3888.6	274.2	11.4
	2012	12-a	9 Jul	Mid	25	0.38	3	1.8	7.8	83.8	1.1
	2012	12-b	19 Jul	Mid	17	0.09	< 2	2.2	24.5	409.3	3.0
	2012	12-c	24 Jul	Mid	21	0.29	< 2	7.2	886.9	691.3	*
	2016	16-a	23 Jul	Mid	24	0.35	2	4.0	*	5.0	161.5
	2016[1]		30 Jul	Mid	28	0.34	3	3.3	0.2	*	*
	2016	16-b	2 Aug	Late	28	0.34	3	0.9	0.4	53.6	1.2
	2017[1]		21 Jul	Mid	4	0.5	< 2	0.5	*	*	*
GS	2007	07-a	22 Jun	Early	14	0.33	2	1.6	0.6	4.6	1.0
	2007	07-b	30 Jun	Early	54	0.19	5	4.5	1.4	11.7	2.9
	2008	08-a	24 Jun	Early	8	0.25	< 2	4.5	1.0	24.8	3.2
	2009	09-a	17 Jul	Mid	75	0.38	100	9.2	5.9	79.6	10.5
	2009	09-b	24 Jul	Mid	36	0.12	< 2	1.4	0.1	15.3	1.9
	2009	09-c	26 Jul	Mid	33	0.39	8	8.0	5.8	80.1	9.4
	2010	10-a	14 Jun	Early	41	0.12	< 2	7.7	11.8	29.0	11.2
	2010	10-b	13 Jul	Mid	66	0.06	< 2	3.9	2.5	13.0	4.2
	2010	10-c	21 Jul	Mid	24	0.10	< 2	1.2	1.2	5.5	1.6
	2012	12-a	9 Jul	Mid	25	0.38	3	1.9	278.8	1125.3	43.6
	2012	12-b	19 Jul	Mid	17	0.09	< 2	1.9	76.0	3882.0	36.6
	2012	12-c	24 Jul	Mid	21	0.29	< 2	4.3	1307.1	7752.1	*
	2016	16-a	23 Jul	Mid	24	0.35	2	1.3	0.1	3.4	0.5
	2016	16-b	30 Jul	Mid	28	0.34	3	5.5	1.4	37.3	7.1
	2017	17-c	20 Jul	Mid	4	0.5	< 2	1.5	3.4	10.2	1.2

Notes:
[1] Incomplete record not displayed on Fig. 2.
* No samples available for this rainfall event

Table S2. Annual fluvial flux (kg) for the different hydrological periods (nival, baseflow, pluvial) for Ptarmigan (PT) and Goose (GS). Total runoff and flux of SS, POC, DOC, and major ions separated into hydrological periods. Note that total SS_{flux} includes POC (POC is a quantifiable component of SS). No hydrological monitoring in 2011, 2013, and 2015

		NIVAL (SNOWMELT)					BASEFLOW (LOW FLOW)					PLUVIAL (RAINFALL)				
Site	Year	Runoff (mm)	SS_{flux} (kg)	POC_{flux} (kg)	DOC_{flux} (kg)	major ion_{flux} (kg)	Runoff (mm)	SS_{flux} (kg)	POC_{flux} (kg)	DOC_{flux} (kg)	major ion_{flux} (kg)	Runoff (mm)	SS_{flux} (kg)	POC_{flux} (kg)	DOC_{flux} (kg)	major ion_{flux} (kg)
PT	2006	17.7			11.6	132.1	2.7									
	2007	12.9	93.5	1.3	8.2	99.1	10.8	49.0	0.7	5.8	99.9	11.6	40.5	0.5	10.6	152.0
	2008	37.4	932.9	12.1	7.4	96.7	1.6	149.9	1.9	0.7	66.4					
	2009	55.5	7874.1	70.6	17.7	947.9	25.8	1223.0	10.7	*	391.9	22.2	5363.5	53.2	21.6	744.4
	2010	141.8	24301.0	300.0	75.3	916.8	0.9	11.9	0.4	0.5	13.9					
	2011															
	2012	49.6	3481.4	29.9	26.8	1233.5	15.8	91.1	1.5	5.8	1131.7	9.0	894.7	12.2	1.1	775.0
	2013															
	2014	31.6	3310.9	47.3	21.4	480.5	2.5	180.0	2.4	0.9	32.2					
	2015															
	2016	20.1	1418.5	19.1	8.5	191.5	8.1	1.4	0.1	<0.1	1.5	5.3	11.4	0.2	6.2	215.0
	2017	34.7	1889.1	21.1	11.3	384.8	2.7	3.1	<0.1	0.1	5.5	0.5				
GS	2006	20.7			12.5	69.9	3.6			2.8	16.6					
	2007	6.3	1.9	0.03	5.0	20.7	8.2	2.4	0.04	5.1	22.9	2.0	0.4	0.01	1.8	6.6
	2008	29.5	9.6	0.20	28.6	225.1	10.0	3.5	0.08	8.3	71.3					
	2009	38.8	13.8	0.15	31.3	301.7	25.4	10.8	0.16	27.0	235.3	17.2	11.7	0.15	19.9	159.7
	2010	78.3	43.0	0.47	73.2	384.9	34.7	18.1	0.24	38.5	161.7					
	2011															
	2012	77.3	19.5	0.30	82.7	5.4	5.4	1.0	0.01	2.1	34.3	8.1	4.5	0.10	6.8	196.3
	2013															
	2014	27.1	10.4	0.15	27.3	129.8	37.3	0.2	<0.01	26.8	308.1					
	2015															
	2016	36.8	14.9	0.24	26.1	222.2	7.3	1.4	0.02	4.7	32.3	6.7	1.4	0.02	7.6	40.8
	2017	68.0	16.3	0.25	37.3	415.8	3.7	3.5	0.05	1.8	13.9	1.5	3.4	0.03	1.2	10.2

* No samples available

Table S3. Runoff-normalized flux (kg mm^{-1}) for the different hydrological periods for Ptarmigan (PT) and Goose (GS). Total runoff for each hydrological period (snowmelt, baseflow, rainfall), runoff-normalized fluxes of SS, POC, DOC, and major ions. SS samples were not available in 2006 and no hydrological monitoring in 2011, 2013, and 2015. Displayed graphically in Fig. 3.

		NIVAL (SNOWMELT)					BASEFLOW (LOW-FLOW)					PLUVIAL (RAINFALL)				
Site	Year	Runoff mm	SS kg mm^{-1}	POC kg mm^{-1}	DOC kg mm^{-1}	major ion kg mm^{-1}	Runoff mm	SS kg mm^{-1}	POC kg mm^{-1}	DOC kg mm^{-1}	major ion kg mm^{-1}	Runoff mm	SS kg mm^{-1}	POC kg mm^{-1}	DOC kg mm^{-1}	major ion kg mm^{-1}
PT	2006	17.7			0.7	7.5	2.7									
	2007	12.9	7.2	0.1	0.6	7.7	7.2	3.7	0.1	0.6	10.2	15.2	4.1	0.1	0.8	11.7
	2008	28.8	267.4	3.3	0.6	16.2	1.6	143.1	1.4	0.4	33.6	2.7	354.2	4.5	0.6	28.2
	2009	55.5	141.9	1.3	0.3	17.1	24.0	48.9	0.4	*	12.7	24.0	225.6	2.2	0.9	34.7
	2010	141.8	171.4	2.1	0.5	6.5	0.9	13.2	0.4	0.6	15.4					
	2011															
	2012	49.6	70.2	0.6	0.5	24.9	13.6	4.9	0.1	0.2	53.1	11.2	82.1	1.1	0.4	105.7
	2013															
	2014	31.6	104.8	1.5	0.7	15.2	2.5	72.0	1.0	0.4	12.9					
	2015															
	2016	20.1	70.6	1.0	0.4	9.5	5.2	2.3	<0.1	1.2	41.3	8.2	0.1	<0.1	0.8	26.2
	2017	34.70	54.4	<0.1	0.3	11.1	2.7	1.1	<0.1	<0.1	2.0	0.4	*	*	*	*
GS	2006	20.7			3.4	0.6	2.8			1.0	5.9					
	2007	6.3	0.3	0.005	3.3	0.8	4.0	0.2	0.003	0.8	3.3	6.1	0.3	0.005	0.6	2.7
	2008	29.5	0.3	0.007	7.6	1.0	5.5	0.5	0.025	0.9	8.5	4.5	0.2	0.007	0.7	5.5
	2009	38.8	0.4	0.004	7.8	0.8	24.0	0.4	0.007	1.0	9.2	18.6	0.6	0.005	1.2	9.4
	2010	78.3	0.5	0.006	4.9	0.9	21.9	0.1	0.005	1.0	5.2	12.8	1.2	0.008	1.3	3.7
	2011															
	2012	77.3	0.3	0.004	8.2	1.1	5.4	0.2	0.002	0.4	6.4	8.1	0.6	0.012	0.8	24.2
	2013															
	2014	27.1	0.4	0.006	4.8	1.0	37.3	<0.1	<0.001	0.7	8.3					
	2015															
	2016	36.8	0.4	0.005	6.0	0.7	7.3	0.2	0.003	0.6	4.4	6.8	0.2	0.003	1.1	6.0
	2017	68.8	0.2	0.004	6.1	0.5	3.7	0.9	0.014	0.5	3.8	1.5	2.3	0.020	0.8	6.8

Notes:

* No available data

Table S4. Annual mean concentrations (mg L^{-1} ± 1 standard deviation) of [DOC], [major ion], [SS], [POC] and the mean ratio of [POC]/[SS] (%) from Ptarmgian (PT) and Goose (GS). No hydrological monitoring in 2011, 2013, and 2015.

**Site**	**Year**	**[DOC] (mg L^{-1})**	**[major ion] (mg L^{-1})**	**[SS] (mg L^{-1})**	**[POC] (mg L^{-1})**	**[POC]/[SS] (%)**
PT	2006	2.8 ± 1.6	34.9 ± 12.9	*	*	*
	2007	3.0 ± 0.7	41.8 ± 9.8	25.5 ± 19.0	0.4 ± 0.2	1.5
	2008	3.5 ± 1.6	119.0 ± 85.0	840.2 ± 814.3	10.5 ± 9.7	1.3
	2009	5.6 ± 3.6[1]	87.7 ± 84.5	514.8 ± 544.1	4.7 ± 5.0	1.2
	2010	2.4 ± 1.5	42.1 ± 20.6	1033.1 ± 1044.4	13.1 ± 12.2	1.2
	2011					
	2012	3.1 ± 2.3	364.2 ± 293.8	338.0 ± 495.7	3.3 ± 3.1	1.4
	2013					
	2014	4.4 ± 2.4[1]	76.7 ± 35.0	439.7 ± 451.6	6.2 ± 6.6	1.4
	2015					
	2016	2.8 ± 2.4	95.5 ± 99.4	214.1 ± 202.3	3.1 ± 2.4	1.3
	2017	1.6 ± 0.3	68.9 ± 30.2	221.5 ± 199.4	2.8 ± 2.5	1.3
GS	2006	3.7 ± 0.6	20.8 ± 3.5	*	*	*
	2007	4.0 ± 0.8	17.2 ± 3.5	1.6 ± 1.5	0.03 ± 0.02	1.5
	2008	5.6 ± 2.5	43.6 ± 11.9	1.9 ± 1.3	0.04 ± 0.03	1.5
	2009	5.7 ± 1.9	48.7 ± 9.8	2.4 ± 2.3	0.03 ± 0.03	1.4
	2010	5.8 ± 1.5	27.1 ± 9.6	2.9 ± 1.9	0.04 ± 0.02	1.3
	2011					
	2012	5.6 ± 2.8	59.0 ± 37.2	2.1 ± 2.5	0.03 ± 0.03	1.3
	2013					
	2014	5.1 ± 2.2	43.5 ± 18.9	0.5 ± 1.2	0.01 ± 0.02	1.4
	2015					
	2016	5.2 ± 1.8	37.6 ± 19.3	1.8 ± 1.7	0.03 ± 0.03	1.4
	2017	3.6 ± 0.7	34.1 ± 4.0	3.8 ± 4.3	0.10 ± 0.10	1.5

Notes:
[1]limited samples collected throghout the hydrological season (2009: n = 11; 2014: n = 18)
* No data available

Table S5. Pearson linear correlation coefficients between [SS], [DOC] and [major ion]. Significant ($p < 0.05$) correlations are bolded. All statistical tests performed using Matlab® (version R2020a; *corrcoef*). No hydrological monitoring in 2011, 2013, and 2015.

Site	Year		[SS]	[DOC]	[major Ion]	Site	Year		[SS]	[DOC]	[major Ion]
PT	2006	[SS]				GS	2006	[SS]			
		[DOC]			0.12			[DOC]			**0.91**
		[major ion]						[major ion]			
	2007	[SS]		-0.09			2007	[SS]		-0.01	
		[DOC]			**0.53**			[DOC]			0.24
		[major ion]						[major ion]			
	2008	[SS]		**-0.55**			2008	[SS]		-0.05	
		[DOC]			-0.27			[DOC]			**0.60**
		[major ion]						[major ion]			
	2009	[SS]		0.04			2009	[SS]		0.04	
		[DOC]			**0.38**			[DOC]			**0.82**
		[major ion]						[major ion]			
	2010	[SS]		-0.38			2010	[SS]		**0.47**	
		[DOC]			-0.24			[DOC]			-0.15
		[major ion]						[major ion]			
	2012	[SS]		-0.31			2012	[SS]		0.18	
		[DOC]			**0.40**			[DOC]			**0.66**
		[major ion]						[major ion]			
	2014	[SS]		0.21			2014	[SS]		0.26	
		[DOC]			**0.67**			[DOC]			**0.39**
		[major ion]						[major ion]			
	2016	[SS]		0.40			2016	[SS]		-0.21	
		[DOC]			0.23			[DOC]			**0.48**
		[major ion]						[major ion]			
	2017	[SS]		-0.39			2017	[SS]		**0.57**	
		[DOC]			-0.07			[DOC]			0.23
		[major ion]						[major ion]			

Table S6. Statistically significant (p <0.05) slope changes in the linear relationship between cumulative DOC_{yield}, major ion_{yield} and SS_{yield} (POC is 1.4 % of SS in both watersheds) for Ptarmigan (PT) and Goose (GS). Significant changes were identified using the double-mass curve approach in combination with one-way analysis of covariance (ANCOVA) using Matlab® (version 2020a; *aoctool*). No hydrological monitoring in 2011, 2013, and 2015. Displayed graphically in Fig. 5**.**

Site	Parameter	Year(s)	Linear slope	ANCOVA $F(d.f._B, d.f._W) = r$	Site	Parameter	Year(s)	Linear Slope	ANCOVA $F(d.f._B, d.f._W) = r$
PT	DOC_{yield}	2006-2008	3.1		GS	DOC_{yield}	2006-2007	3.9	
		2009-2017	2.3	F(1,326) = 65.8			2008-2012	5.6	F(1,204) = 31.1
							2014-2017	3.7	F(1,290) = 24.6
PT	major ion_{yield}	2006-2007	40.9		GS	major ion_{yield}	2006-2007	19.6	
		2008-2009	81.6	F(1,99) = 50.2			2008-2009	44.4	F(1,119) = 1064.0
		2010	30.4	F(1,81) = 251.1			2010	27.2	F(1,102) = 1615.2
		2012	168.5	F(1,66) = 103.7			2012	45.4	F(1,81) = 305.4
		2014-2017	55.0	F(1,203) = 265.7			2014-2017	36.2	F(1,184) = 50.8
PT	SS_{yield}	2007	24.1		GS	SS_{yield}		*no change*	
		2008-2009	724.4	F(1,77) = 20.3					
		2010	986.7	F(1,81) = 18.1					
		2012-2017	309.1	F(1,223) = 434.7					

Notes: ANCOVA results presented as: d.f.B = degrees of freedom between groups, d.f.W = degrees of freedom within groups, r = F-ratio

References

1. Lamoureux, S.F., Lafrenière, M.J., & Favaro, E.A. Erosion dynamics following localized permafrost slope disturbances. *Geophys. Res. Lett.*, **41(15)**, 5499-5505, https://doi.org/10.1002/2014GL060677 (2014).
2. Hickel, W. Seston retention by Whatman GF/C glass-fiber filters. *Marine Ecol. – Progress Series*, **16**, 185-191 (1984).
3. Emmerton, C.A., Lesack, L.F. & Vincent, W.F. Mackenzie River nutrient delivery to the Arctic Ocean and effects of the Mackenzie Delta during open water conditions. *Global Biogeochem. Cycles*, **22**, GB1024, http://doi.org/10.1029/2006GB002856 (2008).
4. Gareis, J.A.L., & Lesack, L.F. Fluxes of particulates and nutrients during hydrologically defined seasonal periods in an ice-affected great Arctic river, the Mackenzie. *Water Resource Research*, **53**, 6109-6132. http://doi.org/10.1002/2017WR020623 (2017).
